# Cyclo­hexyldimethyl­ammonium tetra­hydroxy­penta­borate

**DOI:** 10.1107/S1600536808028869

**Published:** 2008-09-17

**Authors:** Hui Li, Guo-Ming Wang, Shu-Yun Xue, Qiang Liang

**Affiliations:** a Department of Chemistry, Teachers College of Qingdao University, Qingdao, Shandong 266071, People’s Republic of China

## Abstract

The title compound, [C_8_H_18_N]^+^·[B_5_O_6_(OH)_4_]^−^, has been synthesized under mild solvothermal conditions in the presence of *N*,*N*-dimethyl­cyclo­hexyl­amine acting as a template. The structure consists of penta­borate [B_5_O_6_(OH)_4_]^−^ anions connected through O—H⋯O hydrogen bonds into a three-dimensional framework, with large channels along [100], [010] and [001] directions. The [C_8_H_18_N]^+^ cations reside in the channels, inter­acting with the framework through N—H⋯O hydrogen bonds.

## Related literature

For related literature, see: Batsanov *et al.* (1982 ▶); Burns *et al.* (1995 ▶); Chen *et al.* (1995 ▶); Grice *et al.* (1999 ▶); Liu & Li (2006 ▶); Liu *et al.* (2006 ▶); Schubert *et al.* (2000 ▶); Touboul *et al.* (2003 ▶); Wang *et al.* (2004 ▶, 2008*a* ▶,*b* ▶)
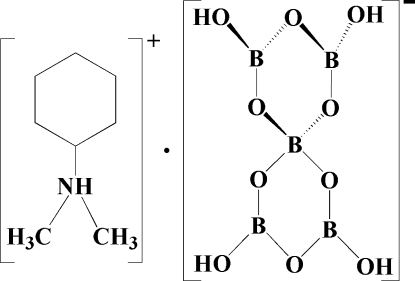


## Experimental

### 

#### Crystal data

C_8_H_18_N^+^·B_5_H_4_O_10_^−^
*M* *_r_* = 346.32
Triclinic, 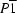

*a* = 8.6971 (4) Å
*b* = 9.8990 (2) Å
*c* = 10.2300 (3) Å
α = 74.591 (3)°
β = 74.442 (2)°
γ = 82.190 (5)°
*V* = 815.98 (5) Å^3^
*Z* = 2
Mo *K*α radiation
μ = 0.12 mm^−1^
*T* = 295 (2) K
0.45 × 0.45 × 0.45 mm

#### Data collection

Bruker SMART APEX area-detector diffractometer
Absorption correction: multi-scan (*SADABS*; Sheldrick, 1996 ▶) *T*_min_ = 0.948, *T*_max_ = 0.949
6623 measured reflections
3318 independent reflections
2536 reflections with *I* > 2σ(*I*)
*R*_int_ = 0.025

#### Refinement

*R*[*F* ^2^ > 2σ(*F* ^2^)] = 0.044
*wR*(*F* ^2^) = 0.119
*S* = 1.08
3318 reflections
218 parameters
H-atom parameters constrained
Δρ_max_ = 0.23 e Å^−3^
Δρ_min_ = −0.29 e Å^−3^

### 

Data collection: *SMART* (Bruker, 2002 ▶); cell refinement: *SAINT-Plus* (Bruker, 2002 ▶); data reduction: *SAINT-Plus*; program(s) used to solve structure: *SHELXS97* (Sheldrick, 2008 ▶); program(s) used to refine structure: *SHELXL97* (Sheldrick, 2008 ▶); molecular graphics: *SHELXTL* (Sheldrick, 2008 ▶); software used to prepare material for publication: *SHELXL97*.

## Supplementary Material

Crystal structure: contains datablocks global, I. DOI: 10.1107/S1600536808028869/mg2056sup1.cif

Structure factors: contains datablocks I. DOI: 10.1107/S1600536808028869/mg2056Isup2.hkl

Additional supplementary materials:  crystallographic information; 3D view; checkCIF report

## Figures and Tables

**Table:** 

B1—O1	1.350 (2)
B1—O5	1.3552 (19)
B1—O2	1.377 (2)
B2—O3	1.341 (2)
B2—O4	1.357 (2)
B2—O2	1.375 (2)
B3—O4	1.452 (2)
B3—O5	1.4651 (19)
B3—O6	1.469 (2)
B3—O7	1.473 (2)
B4—O10	1.346 (2)
B4—O7	1.3491 (19)
B4—O9	1.387 (2)
B5—O8	1.343 (2)
B5—O6	1.3439 (19)
B5—O9	1.388 (2)

**Table:** 

O1—B1—O5	122.22 (15)
O1—B1—O2	117.10 (14)
O5—B1—O2	120.66 (14)
O3—B2—O4	121.91 (16)
O3—B2—O2	117.92 (15)
O4—B2—O2	120.14 (15)
O4—B3—O5	111.21 (12)
O4—B3—O6	108.43 (12)
O5—B3—O6	109.57 (13)
O4—B3—O7	108.58 (13)
O5—B3—O7	108.76 (12)
O6—B3—O7	110.28 (12)
O10—B4—O7	118.13 (15)
O10—B4—O9	121.04 (14)
O7—B4—O9	120.83 (14)
O8—B5—O6	123.78 (14)
O8—B5—O9	115.84 (14)
O6—B5—O9	120.38 (14)

**Table 2:** Hydrogen-bond geometry (Å, °)

*D*—H⋯*A*	*D*—H	H⋯*A*	*D*⋯*A*	*D*—H⋯*A*
O1—H1*A*⋯O5^i^	0.82	1.96	2.7759 (16)	174
O3—H3*A*⋯O4^ii^	0.82	1.99	2.8143 (16)	178
O8—H8*A*⋯O6^iii^	0.82	1.96	2.7816 (15)	179
O10—H10*A*⋯O9^iv^	0.82	2.03	2.8477 (15)	178
N1—H1*D*⋯O7	0.91	1.94	2.8368 (18)	169

Symmetry codes: (i) 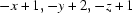; (ii) 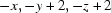; (iii) 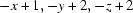; (iv) 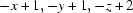.

**Fig. 1.**
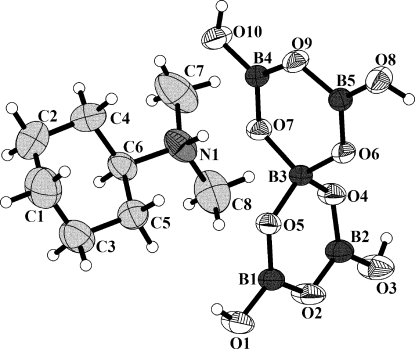
The molecular structure of (I), with displacement ellipsoids drawn at the 50% probability level for non-H atoms.

**Fig. 2.**
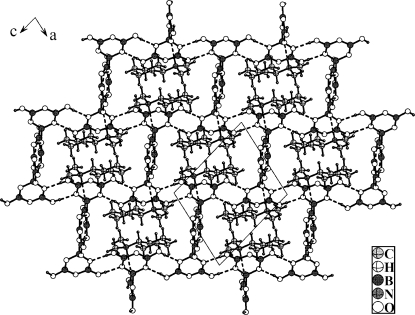
Projection of (I) along b, showing [B5O6(OH)4]- anions linked into a three-dimensional framework, with [C8H18N]+ cations occupying channels. Hydrogen bonds are shown as dashed lines.

## supplementary crystallographic information

### Comment

Borate materials have been receiving particular attention due to their fascinating structural diversities and potential applications in mineralogy and industry (Burns *et al.*, 1995; Chen *et al.*, 1995; Grice *et al.*, 1999; Touboul *et al.*, 2003). From a structural point of view, the ability of B to adopt both BO_3_ and BO_4_ coordination modes, coupled with the tendency of such units to polymerize into a wide range of polyanions, has led to a rapidly growing family of borates. Thus far, numerous inorganic borate materials with alkali metals, alkaline earth metals, rare earths and transition metals have been extensively studied. In contrast, the analogous chemistry of organically templated borates is still relatively undeveloped. To the best of our knowledge, only a few examples with polyanions, such as [B_4_O_5_(OH)_4_] (Batsanov *et al.*, 1982), [B_5_O_6_(OH)_4_] (Wang *et al.*, 2004), [B_7_O_9_(OH)_5_] (Liu & Li, 2006; Liu *et al.*, 2006), [B_9_O_12_(OH)_6_] (Schubert *et al.*, 2000) and [B_14_O_20_(OH)_6_] (Liu *et al.*, 2006), have been reported. The aim of our work is to explore the construction of novel microporous aluminoborates templated by organic agents with different shape and size (Wang *et al.*, 2008a,b). Unexpectedly, the title compound, (I), was isolated, a new organically templated pentaborate.

As shown in Fig. 1, the asymmetric unit of (I) contains one [B_5_O_6_(OH)_4_]^-^ anion and one [C_8_H_18_N]^+^ cation. The anionic [B_5_O_6_(OH)_4_]^-^ polyanion is composed of two common B_3_O_3_ rings, each containing two BO_3_ triangles and one BO_4_ tetrahedron. The B—O bond distances lie in the range 1.341 (2)–1.388 (2) Å for the BO_3_ triangles (B1, B2, B4 and B5) and 1.452 (2)–1.473 (2) Å for the B(3)O_4_ tetrahedron, in good agreement with those reported previously for other borate compounds. The O—B—O bond angles lie in the range 115.8 (2)–123.7 (2) ° for the triangles and 108.4 (2)–111.2 (2) ° for the tetrahedron. The anionic [B_5_O_6_(OH)_4_]^-^ groups are connected to each other through intermolecular O—H···O hydrogen bonds, forming a three-dimensional framework with large channels along [100], [010] and [001] directions. The [C_8_H_18_N]^+^ cations reside in these channels, interacting with the framework through N—H···O hydrogen bonds (Fig. 2).

### Experimental

A mixture of H_3_BO_3_ (0.186 g), Al_2_O_3_ (0.104 g), *N*,*N*-dimethylcyclohexylamine (0.75 ml), pyridine (4.4 ml) and H_2_O (0.50 ml) was sealed in a Teflon-lined steel autoclave, heated at 453 K for 8 days, and then cooled to room temperature. The homogeneous product consisting of large colorless block-shaped crystals was separated from the solution by filtration, washed with distilled water, and then dried in air.

### Refinement

All H atoms were positioned geometrically and treated as riding atoms: O—H = 0.82 Å, N—H = 0.91 Å and C—H = 0.96–0.98 Å with *U*_iso_(H) = 1.2–1.5*U*_eq_(parent atoms).

### Crystal data

**Table:**

C_8_H_18_N^+^·B_5_H_4_O^10^^−^	*Z* = 2
*M**_r_* = 346.32	*F*(000) = 364
Triclinic, *P*1	*D*_x_ = 1.410 Mg m^−^^3^
Hall symbol: -P 1	Mo *K*α radiation, λ = 0.71073 Å
*a* = 8.6971 (4) Å	Cell parameters from 6623 reflections
*b* = 9.8990 (2) Å	θ = 2.1–26.5°
*c* = 10.2300 (3) Å	µ = 0.12 mm^−^^1^
α = 74.591 (3)°	*T* = 295 K
β = 74.442 (2)°	Block, colorless
γ = 82.190 (5)°	0.45 × 0.45 × 0.45 mm
*V* = 815.98 (5) Å^3^	

### Data collection

**Table:**

Bruker SMART APEX area-detector diffractometer	3318 independent reflections
Radiation source: fine-focus sealed tube	2536 reflections with *I* > 2σ(*I*)
graphite	*R*_int_ = 0.026
φ and ω scans	θ_max_ = 26.5°, θ_min_ = 2.1°
Absorption correction: multi-scan (SADABS; Sheldrick, 1996)	*h* = −10→10
*T*_min_ = 0.949, *T*_max_ = 0.949	*k* = −12→12
6623 measured reflections	*l* = −12→12

### Refinement

**Table:**

Refinement on *F*^2^	Secondary atom site location: difference Fourier map
Least-squares matrix: full	Hydrogen site location: inferred from neighbouring sites
*R*[*F*^2^ > 2σ(*F*^2^)] = 0.044	H-atom parameters constrained
*wR*(*F*^2^) = 0.119	*w* = 1/[σ^2^(*F*_o_^2^) + (0.0587*P*)^2^ + 0.0744*P*] where *P* = (*F*_o_^2^ + 2*F*_c_^2^)/3
*S* = 1.08	(Δ/σ)_max_ < 0.001
3318 reflections	Δρ_max_ = 0.23 e Å^−^^3^
218 parameters	Δρ_min_ = −0.29 e Å^−^^3^
0 restraints	Extinction correction: SHELXL, Fc^*^=kFc[1+0.001xFc^2^λ^3^/sin(2θ)]^-1/4^
Primary atom site location: structure-invariant direct methods	Extinction coefficient: 0.058 (6)

### Special details

**Table:**

Geometry. All e.s.d.'s (except the e.s.d. in the dihedral angle between two l.s. planes) are estimated using the full covariance matrix. The cell e.s.d.'s are taken into account individually in the estimation of e.s.d.'s in distances, angles and torsion angles; correlations between e.s.d.'s in cell parameters are only used when they are defined by crystal symmetry. An approximate (isotropic) treatment of cell e.s.d.'s is used for estimating e.s.d.'s involving l.s. planes.
Refinement. Refinement of *F*^2^ against ALL reflections. The weighted *R*-factor *wR* and goodness of fit *S* are based on *F*^2^, conventional *R*-factors *R* are based on *F*, with *F* set to zero for negative *F*^2^. The threshold expression of *F*^2^ > σ(*F*^2^) is used only for calculating *R*-factors(gt) *etc*. and is not relevant to the choice of reflections for refinement. *R*-factors based on *F*^2^ are statistically about twice as large as those based on *F*, and *R*- factors based on ALL data will be even larger.

### Fractional atomic coordinates and isotropic or equivalent isotropic displacement parameters (Å^2^)

**Table:**

	*x*	*y*	*z*	*U*_iso_*/*U*_eq_	
B1	0.3071 (2)	1.10774 (18)	0.59941 (19)	0.0342 (4)	
B2	0.1056 (2)	1.1184 (2)	0.8073 (2)	0.0372 (4)	
B3	0.3133 (2)	0.92080 (17)	0.81503 (17)	0.0296 (4)	
B4	0.3702 (2)	0.66200 (18)	0.88838 (18)	0.0309 (4)	
B5	0.5114 (2)	0.81317 (18)	0.95617 (18)	0.0311 (4)	
O1	0.35979 (15)	1.16205 (12)	0.46086 (12)	0.0477 (3)	
H1A	0.4376	1.1133	0.4291	0.072*	
O2	0.17524 (14)	1.17636 (12)	0.66942 (12)	0.0480 (3)	
O3	−0.02096 (16)	1.18988 (13)	0.87215 (13)	0.0555 (4)	
H3A	−0.0641	1.1391	0.9470	0.083*	
O4	0.16229 (13)	0.99089 (11)	0.87271 (11)	0.0362 (3)	
O5	0.37677 (12)	0.98883 (11)	0.66764 (10)	0.0328 (3)	
O6	0.42790 (13)	0.92583 (10)	0.89591 (11)	0.0334 (3)	
O7	0.28642 (13)	0.77398 (10)	0.82604 (11)	0.0331 (3)	
O8	0.61961 (15)	0.82028 (12)	1.02584 (13)	0.0454 (3)	
H8A	0.6045	0.8953	1.0486	0.068*	
O9	0.48965 (13)	0.67959 (11)	0.94784 (12)	0.0375 (3)	
O10	0.33377 (15)	0.53291 (11)	0.89197 (13)	0.0453 (3)	
H10A	0.3859	0.4736	0.9386	0.068*	
C2	0.2698 (4)	0.5351 (3)	0.4335 (3)	0.0907 (9)	
H2A	0.3327	0.4461	0.4360	0.109*	
H2B	0.1854	0.5349	0.3880	0.109*	
C1	0.3752 (4)	0.6525 (3)	0.3495 (3)	0.0884 (8)	
H1B	0.4659	0.6475	0.3892	0.106*	
H1C	0.4156	0.6425	0.2542	0.106*	
C4	0.1958 (3)	0.5485 (2)	0.5814 (2)	0.0685 (6)	
H4A	0.1248	0.4738	0.6301	0.082*	
H4B	0.2793	0.5392	0.6304	0.082*	
C3	0.2836 (3)	0.7915 (3)	0.3491 (2)	0.0677 (6)	
H3B	0.2010	0.8008	0.2990	0.081*	
H3C	0.3552	0.8659	0.3005	0.081*	
C5	0.2074 (3)	0.8069 (2)	0.4963 (2)	0.0552 (5)	
H5A	0.2908	0.8097	0.5421	0.066*	
H5B	0.1428	0.8952	0.4922	0.066*	
C6	0.1036 (2)	0.6880 (2)	0.58154 (18)	0.0464 (4)	
H6A	0.0141	0.6920	0.5391	0.056*	
C7	−0.0497 (3)	0.5880 (3)	0.8280 (3)	0.0899 (9)	
H7A	−0.0881	0.6078	0.9185	0.135*	
H7B	0.0219	0.5050	0.8341	0.135*	
H7D	−0.1386	0.5732	0.7956	0.135*	
C8	−0.0711 (3)	0.8397 (3)	0.7322 (3)	0.0774 (7)	
H8B	−0.1092	0.8485	0.8270	0.116*	
H8E	−0.1603	0.8354	0.6953	0.116*	
H8C	−0.0124	0.9194	0.6767	0.116*	
N1	0.03619 (19)	0.70824 (19)	0.72858 (16)	0.0540 (4)	
H1D	0.1208	0.7174	0.7614	0.065*	

### Atomic displacement parameters (Å^2^)

**Table:**

	*U*^11^	*U*^22^	*U*^33^	*U*^12^	*U*^13^	*U*^23^
B1	0.0377 (10)	0.0291 (9)	0.0329 (9)	0.0001 (8)	−0.0099 (8)	−0.0021 (7)
B2	0.0364 (10)	0.0328 (10)	0.0367 (10)	0.0043 (8)	−0.0081 (8)	−0.0027 (8)
B3	0.0345 (9)	0.0252 (8)	0.0286 (9)	0.0013 (7)	−0.0110 (7)	−0.0038 (7)
B4	0.0346 (9)	0.0268 (9)	0.0308 (9)	−0.0004 (7)	−0.0100 (7)	−0.0050 (7)
B5	0.0318 (9)	0.0298 (9)	0.0316 (9)	0.0026 (7)	−0.0096 (7)	−0.0075 (7)
O1	0.0515 (8)	0.0430 (7)	0.0336 (6)	0.0102 (6)	−0.0047 (5)	0.0038 (5)
O2	0.0495 (8)	0.0379 (7)	0.0381 (7)	0.0151 (5)	−0.0026 (5)	0.0048 (5)
O3	0.0515 (8)	0.0468 (8)	0.0467 (7)	0.0198 (6)	0.0006 (6)	0.0008 (6)
O4	0.0361 (6)	0.0325 (6)	0.0319 (6)	0.0059 (5)	−0.0056 (5)	−0.0010 (5)
O5	0.0364 (6)	0.0296 (6)	0.0284 (6)	0.0036 (4)	−0.0073 (4)	−0.0037 (4)
O6	0.0422 (6)	0.0247 (5)	0.0361 (6)	0.0024 (5)	−0.0179 (5)	−0.0062 (4)
O7	0.0372 (6)	0.0274 (6)	0.0370 (6)	−0.0002 (5)	−0.0177 (5)	−0.0037 (4)
O8	0.0540 (8)	0.0337 (6)	0.0605 (8)	0.0078 (5)	−0.0354 (6)	−0.0155 (5)
O9	0.0439 (7)	0.0248 (6)	0.0496 (7)	0.0050 (5)	−0.0261 (5)	−0.0076 (5)
O10	0.0546 (8)	0.0262 (6)	0.0614 (8)	−0.0016 (5)	−0.0320 (6)	−0.0036 (5)
C2	0.130 (2)	0.0621 (16)	0.0858 (19)	0.0030 (16)	−0.0256 (18)	−0.0321 (14)
C1	0.0910 (19)	0.101 (2)	0.0638 (15)	0.0082 (16)	−0.0015 (14)	−0.0295 (15)
C4	0.0907 (17)	0.0440 (12)	0.0684 (15)	−0.0043 (11)	−0.0254 (13)	−0.0035 (10)
C3	0.0751 (15)	0.0731 (15)	0.0487 (12)	−0.0154 (12)	−0.0111 (11)	−0.0032 (11)
C5	0.0682 (13)	0.0471 (11)	0.0508 (11)	−0.0127 (10)	−0.0154 (10)	−0.0073 (9)
C6	0.0485 (11)	0.0532 (11)	0.0407 (10)	−0.0104 (9)	−0.0183 (8)	−0.0055 (8)
C7	0.0835 (18)	0.125 (2)	0.0532 (14)	−0.0480 (17)	−0.0113 (13)	0.0068 (14)
C8	0.0516 (13)	0.110 (2)	0.0765 (16)	0.0118 (13)	−0.0205 (12)	−0.0362 (15)
N1	0.0436 (9)	0.0767 (12)	0.0442 (9)	−0.0127 (8)	−0.0178 (7)	−0.0071 (8)

### Geometric parameters (Å, °)

**Table:**

B1—O1	1.350 (2)	C1—C3	1.492 (4)
B1—O5	1.3552 (19)	C1—H1B	0.9700
B1—O2	1.377 (2)	C1—H1C	0.9700
B2—O3	1.341 (2)	C4—C6	1.500 (3)
B2—O4	1.357 (2)	C4—H4A	0.9700
B2—O2	1.375 (2)	C4—H4B	0.9700
B3—O4	1.452 (2)	C3—C5	1.513 (3)
B3—O5	1.4651 (19)	C3—H3B	0.9700
B3—O6	1.469 (2)	C3—H3C	0.9700
B3—O7	1.473 (2)	C5—C6	1.510 (3)
B4—O10	1.346 (2)	C5—H5A	0.9700
B4—O7	1.3491 (19)	C5—H5B	0.9700
B4—O9	1.387 (2)	C6—N1	1.517 (2)
B5—O8	1.343 (2)	C6—H6A	0.9800
B5—O6	1.3439 (19)	C7—N1	1.484 (3)
B5—O9	1.388 (2)	C7—H7A	0.9600
O1—H1A	0.8200	C7—H7B	0.9600
O3—H3A	0.8200	C7—H7D	0.9600
O8—H8A	0.8200	C8—N1	1.497 (3)
O10—H10A	0.8200	C8—H8B	0.9600
C2—C1	1.506 (4)	C8—H8E	0.9600
C2—C4	1.510 (3)	C8—H8C	0.9600
C2—H2A	0.9700	N1—H1D	0.9100
C2—H2B	0.9700		
			
O1—B1—O5	122.22 (15)	C6—C4—H4A	109.5
O1—B1—O2	117.10 (14)	C2—C4—H4A	109.5
O5—B1—O2	120.66 (14)	C6—C4—H4B	109.5
O3—B2—O4	121.91 (16)	C2—C4—H4B	109.5
O3—B2—O2	117.92 (15)	H4A—C4—H4B	108.1
O4—B2—O2	120.14 (15)	C1—C3—C5	111.33 (19)
O4—B3—O5	111.21 (12)	C1—C3—H3B	109.4
O4—B3—O6	108.43 (12)	C5—C3—H3B	109.4
O5—B3—O6	109.57 (13)	C1—C3—H3C	109.4
O4—B3—O7	108.58 (13)	C5—C3—H3C	109.4
O5—B3—O7	108.76 (12)	H3B—C3—H3C	108.0
O6—B3—O7	110.28 (12)	C6—C5—C3	112.21 (17)
O10—B4—O7	118.13 (15)	C6—C5—H5A	109.2
O10—B4—O9	121.04 (14)	C3—C5—H5A	109.2
O7—B4—O9	120.83 (14)	C6—C5—H5B	109.2
O8—B5—O6	123.78 (14)	C3—C5—H5B	109.2
O8—B5—O9	115.84 (14)	H5A—C5—H5B	107.9
O6—B5—O9	120.38 (14)	C4—C6—C5	110.93 (18)
B1—O1—H1A	109.5	C4—C6—N1	111.82 (15)
B2—O2—B1	119.89 (13)	C5—C6—N1	109.11 (15)
B2—O3—H3A	109.5	C4—C6—H6A	108.3
B2—O4—B3	123.53 (13)	C5—C6—H6A	108.3
B1—O5—B3	123.25 (13)	N1—C6—H6A	108.3
B5—O6—B3	124.60 (12)	N1—C7—H7A	109.5
B4—O7—B3	123.84 (12)	N1—C7—H7B	109.5
B5—O8—H8A	109.5	H7A—C7—H7B	109.5
B4—O9—B5	119.28 (12)	N1—C7—H7D	109.5
B4—O10—H10A	109.5	H7A—C7—H7D	109.5
C1—C2—C4	112.3 (2)	H7B—C7—H7D	109.5
C1—C2—H2A	109.1	N1—C8—H8B	109.5
C4—C2—H2A	109.1	N1—C8—H8E	109.5
C1—C2—H2B	109.1	H8B—C8—H8E	109.5
C4—C2—H2B	109.1	N1—C8—H8C	109.5
H2A—C2—H2B	107.9	H8B—C8—H8C	109.5
C3—C1—C2	110.5 (2)	H8E—C8—H8C	109.5
C3—C1—H1B	109.6	C7—N1—C8	108.9 (2)
C2—C1—H1B	109.6	C7—N1—C6	114.16 (19)
C3—C1—H1C	109.6	C8—N1—C6	112.63 (16)
C2—C1—H1C	109.6	C7—N1—H1D	106.9
H1B—C1—H1C	108.1	C8—N1—H1D	106.9
C6—C4—C2	110.61 (18)	C6—N1—H1D	106.9

### Hydrogen-bond geometry (Å, °)

**Table:**

*D*—H···*A*	*D*—H	H···*A*	*D*···*A*	*D*—H···*A*
O1—H1A···O5^i^	0.82	1.96	2.7759 (16)	174.
O3—H3A···O4^ii^	0.82	1.99	2.8143 (16)	178.
O8—H8A···O6^iii^	0.82	1.96	2.7816 (15)	179.
O10—H10A···O9^iv^	0.82	2.03	2.8477 (15)	178.
N1—H1D···O7	0.91	1.94	2.8368 (18)	169.

Symmetry codes: (i) −*x*+1, −*y*+2, −*z*+1; (ii) −*x*, −*y*+2, −*z*+2; (iii) −*x*+1, −*y*+2, −*z*+2; (iv) −*x*+1, −*y*+1, −*z*+2.
